# HIV Late Presenters in Asia: Management and Public Health Challenges

**DOI:** 10.1155/2023/9488051

**Published:** 2023-06-14

**Authors:** Chen Seong Wong, Lyu Wei, Yeon-Sook Kim

**Affiliations:** ^1^National Centre for Infectious Diseases, Singapore; ^2^Department of Infectious Diseases, Tan Tock Seng Hospital, Singapore; ^3^Department of Medicine, Yong Loo Lin School of Medicine, National University of Singapore, Singapore; ^4^Department of Infectious Diseases, Peking Union Medical College Hospital in Beijing, Beijing, China; ^5^Division of Infectious Diseases, Department of Internal Medicine, Chungnam National University School of Medicine, Daejeon, Republic of Korea

## Abstract

Many individuals are diagnosed with human immunodeficiency virus (HIV) infection at an advanced stage of illness and are considered late presenters. We define late presentation as a CD4 cell count below 350 cells/mm^3^ at the time of HIV diagnosis, or presenting with an AIDS-defining illness regardless of CD4 count. Across Asia, an estimated 34–72% of people diagnosed with HIV are late presenters. HIV late presenters generally have a higher disease burden and higher comorbidity such as opportunistic infections than those who are diagnosed earlier. They also have a higher mortality rate and generally exhibit poorer immune recovery following combined antiretroviral therapy (cART). As such, late HIV presentation leads to increased resource burden and costs to healthcare systems. HIV late presentation also poses an increased risk of community transmission since the transmission rate from people unaware of their HIV status is approximately 3.5 times higher than that of early presenters. There are several factors which contribute to HIV late presentation. Fear of stigmatisation and discrimination are significant barriers to both testing and accessing treatment. A lack of perceived risk and a lack of knowledge by individuals also contribute to late presentation. Lack of referral for testing by healthcare providers is another identified barrier in China and may extend to other regions across Asia. Effective strategies are still needed to reduce the incidence of late presentation across Asia. Key areas of focus should be increasing community awareness of the risk of HIV, reducing stigma and discrimination in testing, and educating healthcare professionals on the need for early testing and on the most effective ways to engage with people living with HIV. Recent initiatives such as intensified patient adherence support programs and HIV self-testing also have the potential to improve access to testing and reduce late diagnosis.

## 1. Introduction

In 2021, there were approximately 38.4 million people across the globe with HIV, of which 1.7 million were children (0–14 years old) and 54% were female [1]. Although the incidence of HIV remains high, there has been considerable progress in the fight against HIV/acquired immunodeficiency syndrome (AIDS). Since 2010, the global incidence of new HIV infections has decreased by approximately 32%, and AIDS-related deaths have decreased by 52% [1]. These improvements may be attributed to the dramatic increase in the number of people receiving HIV treatment in resource-limited countries [1]. Despite the improvements in global transmission and mortality rates, Asia and the Pacific still have a significant HIV burden. In 2021, the region had the second-highest number of people living with HIV (6.0 million) and 260,000 newly acquired infections that year [1]. Moreover, the proportion of people who are diagnosed late (termed late presentation) is considerable.

There have been many different published definitions for HIV late presentation over the last twenty years [2]. Initiatives have moved towards a harmonised definition, with the consensus definition of late presentation being defined as a CD4 cell count below 350 cells/mm^3^ at the time of HIV diagnosis or presenting with an AIDS-defining event regardless of CD4 count [2]. Advanced HIV disease is defined as a CD4 count below 200 cells/mm^3^ or presenting with an AIDS-defining event, regardless of the CD4 cell count [2].

HIV/AIDS late presentation poses serious health concerns, including increased risk of onward transmission, heightened susceptibility to opportunistic infections, and greater mortality [3, 4]. Late presenters are also at an increased risk of treatment resistance and have higher healthcare costs [5, 6]. This article describes HIV late presentation in Asia, including management and public health challenges associated with HIV late presentation.

## 2. Epidemiology of HIV Late Presenters in Asia

A considerable proportion of people diagnosed with HIV in the Asia-Pacific region are late presenters. In the period of 2003–2012, the TAHOD (TREAT Asia HIV Observational Database) study of 3744 people living with HIV (PLWH) found that 72% of new diagnoses in the Asia-Pacific region were advanced HIV late presenters (with a CD4 count <200 cells/mm^3^ or an AIDS-defining event within three months of first positive HIV test) [7]. Using a similar definition (AIDS or World Health Organisation (WHO) stage 3 or 4 HIV/AIDS, or had a CD4 cell count <200 cells/mm^3^ at the time of diagnosis), a large study of 528,230 Chinese HIV PLWH found only 34% of cases between 2006 and 2014 to be late presenters [8]. Furthermore, a meta-analysis of 39 studies determined the prevalence of late presentation to HIV/AIDS care was 43.26% in China [9]. In Singapore, the late presenter rate between 2012 and 2017 was estimated at 45% (CD4 count <200 cells/mm^3^ at the time of diagnosis or an AIDS-defining illness at diagnosis or within one year of HIV diagnosis) [10]. Although the prevalence varies regionally, late presentation remains high across Asia, and efforts to raise awareness of HIV/AIDS and encourage early testing are required.

Europe, the United States, and Australia have reported lower proportions of HIV late presenters despite using a higher cutoff of CD4 count <350 cells/mm^3^ or an AIDS-event at HIV diagnosis. Australia reported that 36% of new HIV cases were late presenters in 2017 [11], while European estimates stand at approximately 47–57% late presenters [3, 5, 12]. The prevalence of late presenters in Asia using the cutoff of CD4 count <350 cells/mm^3^ remains unclear; however, the study by Tang et al. [8] suggests that it may be higher than that in Western countries.

## 3. Clinical and Social Implications of HIV Late Presentation

HIV late presentation leads to increased resource burden and costs to healthcare systems. The cost of medical care for late presenters is more than double of that of early presenters [6]. Late presentation for treatment can also impact the prognosis for PLWH. Late presenters are at increased risk of clinical events such as opportunistic infections, noninfectious morbidity, and death [3, 4], exhibit poorer immune recovery following combined antiretroviral therapy (ART), and higher prevalence of ART toxicity [5]. They also have a 3.5-fold greater chance of developing an opportunistic infection than nonlate presenters [13]. Survival rates are also lower in late presenters compared to nonlate presenters; however, the impact of late presentation on overall mortality has been shrinking over the years; the 5-year survival rate between 2004 and 2009 for late presenters and nonlate presenters was similar (92% and 97%, respectively) [14]. The gradual increase in survival rate for late presenters over the years is likely due to the improved effectiveness of antiretroviral therapeutics and with the earlier initiation of antiretroviral therapy recommended in treatment guidelines. Despite these improvements in survival rates, late presentation remains a risk for communities. The number of HIV late presenters has been correlated with community viral load [15], and the transmission rate from people unaware of their HIV status is approximately 3.5 times higher than early presenters [15]. As such, late HIV presentation increases the risk of community HIV transmission due to a higher viral load. Early HIV diagnosis is important to achieving earlier viral reduction through treatment, thus reducing risk of transmission.

As a part of its ambitious goal to end HIV/AIDS as a public health threat, the Joint United Nations Programme on HIV/AIDS (UNAIDS) issued its 90-90-90 global target to be reached by 2020; this target aimed to ensure that 90% of HIV-positive people know their HIV status, that 90% of people with a diagnosed HIV infection receive adequate ART, and that 90% of the people receiving ART have viral suppression. Although there have been significant gains in all these areas over the years, the 90-90-90 target was not achieved. By 2020, only 81% of people with HIV know their HIV status, only 67% were on ART, and only 59% had suppressed viral loads [16]. Late presentation is likely to be a contributing factor as to why this target was not achieved. A new UNAIDS target of 95-95-95 by 2030 was recently issued.

## 4. Reasons for Late Presentation to Care

Across Asia, key populations have been identified at a greater risk of late presentation. The TAHOD study, a multicentre observational cohort study that assessed regional HIV treatment outcomes in the Asia-Pacific region, found that older PLWH (≥50 years old) were more likely to be late presenters. Injecting drug users were more likely to be late presenters than those with heterosexual HIV exposure as the main risk factor for transmission; however, heterosexual HIV exposure individuals were more likely to be late presenters than homosexual HIV exposure individuals. Furthermore, males were more likely to be late presenters than females [7]. In Singapore, older age at diagnosis, lower education level, detection via medical care, and heterosexual transmission were identified as risk factors [10]. These Asia-Pacific findings corresponded with findings in Western Europe which also identified older age, male gender, heterosexual transmission, and injecting drug use as factors associated with late presentations [17–20]. Region of origin is also a risk factor for late presentation in regions with higher migration; the proportion of late presenters in Australia and European regions is higher amongst people born in Southeast Asia, sub-Saharan Africa, and Central America [11, 18–20].

Barriers to HIV testing amongst PLWH in resource-rich regions such as Singapore, the United States, Canada, Europe, and Australia are a lack of perceived risk, a lack of knowledge, fear of the diagnosis, stigma, discrimination, and fear regarding lack of confidentiality [10, 21–23]. Late presentation to care in resource-rich environments appears to be strongly driven by late HIV testing due to low-risk perception and the lack of awareness about HIV [10, 24]. For example, in a Swiss HIV cohort study of 680 late presenters, the main reasons identified for late HIV testing were “did not feel at risk” (72%), “did not feel ill” (65%), and “did not know the symptoms of HIV” (51%) [24]. In a Singaporean cross-sectional study of 2188 people, the most common reason (73.7%) for no previous HIV testing was “not necessary to test” [10]. HIV-related stigma may manifest as a wide spectrum of discriminatory behaviour towards people living with HIV, including refusal of care by healthcare professionals, disclosure of diagnosis, reduced access to social services, and discriminatory speech or other behaviour [25]. HIV stigma may also be structural; in many countries in the region, laws criminalising the transmission of HIV are still in force, many of which enforce a heavy penalty even in situations of inadvertent transmission or failure to disclose one's HIV serostatus to one's sexual partners [26]. These manifestations of stigma, whether anticipated or experienced (or both), have been shown to have a range of deleterious effects on the lives of people living with HIV in Asia, including delayed access to testing services, poorer linkage to specialist care, suboptimal adherence to daily antiretroviral therapy, and an increased risk of loss to follow-up [27, 28]. In resource-limited environments across Asia and Africa, fear of stigmatisation is a significant barrier to both testing and accessing treatment [8, 29–34]. In China, a study of 528,234 individuals diagnosed between 2006 and 2014 found that most PLWH did not seek testing until symptoms emerged because of anticipated HIV-associated stigma and a fear of societal discrimination [8]. Moreover, nearly half of Chinese healthcare providers believe that treating people in high-risk populations would scare non-PLWH patients away due to stigmatising attitudes, and one-third were concerned that they would become infected with HIV [35]. In South Asia, there are also significant stigmatising attitudes in the general population, including daily harassment and abuse, which cause mental health erosion and social isolation. It is important to note that stigma and discrimination directed against key populations most affected by HIV, including men who have sex with men, transgender individuals, people who use drugs and sex workers, also have been implicated in poorer HIV-related outcomes. In settings where they are discriminated against, members of key populations are less likely to test regularly for HIV, access preventative care such as pre-exposure prophylaxis, and are less likely to have access to risk reduction interventions such as substance recovery programmes and safer sex education [36–39]. Discriminatory treatment and marginalisation by policy makers and healthcare providers further deters individuals from accessing early screening and treatment [40].

Lack of referral for testing is an identified barrier in China and may extend to other regions across Asia. A nationwide cross-sectional survey of 3580 community healthcare providers found that only one-quarter of staff would provide healthcare testing when requested by a patient [35]. Staff who would offer HIV testing tended to be younger, held tertiary qualifications, had previous HIV training, or were trained as a doctor. Most staff were concerned about reimbursement and half cited lack of training as a major barrier [35], thus highlighting that better staff training and financial reimbursement may help increase HIV testing across China.

HIV self-testing (HIVST) is a relatively new intervention that has the potential to improve access to testing and reduce late diagnosis. HIVST allows individuals to collect their own samples, process the test, and analyse the result themselves [41]. HIVST has been shown to increase testing in key populations such as female sex workers and men who have sex with men [42]. HIVST responds to some service access barriers, particularly for minority ethnic populations who are likely to experience greater anxiety and discomfort in clinic waiting rooms [43]. This is of particular concern in the countries discussed in this paper, which have relatively homogenous ethnic compositions and yet where minority populations are represented in the HIV-positive communities (for example, people of Malay and Indian ethnicity in majority Chinese Singapore).

Despite a strong uptake into public health policy, HIVST has not yet been effectively implemented and scaled up in the Asia-Pacific region [44]. However, many countries in Asia have introduced self-testing in recent years, particularly in the setting of demonstration studies or implementation trials prior to wider rollout [45–47]. Adoption of HIV self-testing, even where available, varies throughout the region. A study in Singapore found that the fear of a positive diagnosis and a perceived lack of confidentiality contributed to barriers to HIV self-testing; a study from the Philippines showed that despite the increased convenience of tests, there were concerns of lack of privacy associated with purchasing and delivery of self-test kits [48, 49]. Overall, barriers to HIV self-test implementation and adoption are multidimensional and setting-specific and deserve more study.

Overall, early detection of HIV is critical in slowing HIV spread and in reducing the risk of morbidity and mortality in infected people [50]. As such, groups at high risk of late diagnosis should be targeted in public health campaigns and encouraged to seek earlier treatment. HIV-related stigma not only affects people with HIV but also those seeking testing. Key factors which will likely reduce the incidence of late HIV presenters are: increasing community awareness of the risk of HIV, reducing stigma and discrimination in testing, and educating healthcare professionals on the need for early testing and on the most effective ways to engage with PLWH.

## 5. Opportunistic Infections in Late Presenters

Opportunistic infections commonly affect people with HIV with very low CD4 counts (<200 cells/mm^3^) due to progressive immune suppression [51]. For example, the rate of bacterial enteric infections is at least 10 times higher in adults with HIV than the general population [52]. Although there are regional differences in the prevalence of opportunistic infections, the three most common opportunistic infections in developing Asian countries are *Pneumocystis jirovecii* pneumonia (PJP), *Mycobacterium tuberculosis* (TB), and cryptococcal meningitis [53, 54]. The treatment of opportunistic infections in HIV is tailored to the infecting organism [51]; however, drug-drug interactions and drug toxicity present challenges when treating people with HIV who have opportunistic infections [55].

There are no effective treatments for many opportunistic infections such as JC virus-associated progressive multifocal leukoencephalopathy, making ART initiation the best defence as it promotes immune reconstitution [56]. Rapid initiation of ART therapy is recommended postdiagnosis, with some exceptions. WHO guidelines recommend delaying ART initiation if individuals with advanced HIV present clinical signs of cryptococcal meningitis or tuberculosis (TB) [57]. Singapore guidelines also recommend delaying ART initiation in the case of cytomegalovirus (CMV) retinitis or central nervous system (CNS) opportunistic infections, at least until specific therapy has been initiated for these infections, and clinical improvement is observed [56]. Although clinical studies show that early initiation of ART in individuals with TB significantly reduces mortality, the risk of immune reconstitution inflammatory syndrome (IRIS) from early initiation is increased [58–60]. As such, Singapore guidelines recommend that ART should be started within two weeks of TB treatment initiation for PLWH with CD4 count <50 cells/mm^3^ but started within two to eight weeks of TB treatment initiation if the CD4 count is ≥50 cells/mm^3^ [56]. Early ART initiation in PLWH with cryptococcal meningitis or tuberculosis meningitis may also lead to IRIS-related complications, including mortality [61, 62]. As such, it is recommended that ART is delayed in PLWH with TB or cryptococcal meningitis until after the individual begins treatment for the opportunistic infection and shows clinical improvement [56]. Individuals with CMV retinitis are also at risk of IRIS-related complications from early ART initiation which can lead to blindness. As such, CMV retinitis treatment must be initiated before ART initiation; the timing of ART initiation should be individualised and should be done in consultation with ophthalmologists experienced in CMV retinitis management [56].

Other management strategies for opportunistic infections, including screening and vaccinations, can also prevent or reduce opportunistic infections [63]. Antibiotic prophylaxis in addition to ART is recommended for PLWH with severely low CD4 counts [52]. Generally, the screening and management of opportunistic infections in advanced HIV disease is performed according to the WHO guidelines [52]. Sputum Xpert MTB/RIF for TB is recommended in symptomatic PLWH with any CD4 counts and urine LF-LAM in PLWH with CD4 ≤ 100 cells/mm^3^ in case of symptoms and signs of TB [57]. Cryptococcal antigen (CrAg) screening is recommended in PLWH with CD4 ≤ 100 cells/mm^3^. These tests are not available in all countries, and therefore, the application of the WHO guideline for advanced HIV disease is limited in AP region. Korean guidelines recommend screening for other opportunistic infections such as hepatitis B, hepatitis C, toxoplasmosis, as well as sexually transmitted diseases such as syphilis, gonorrhoea, and chlamydia [55]. The WHO also recommends cotrimoxazole prophylaxis for CD4 ≤ 350 cells/mm^3^ or WHO clinical stage 3 or 4 event, or in regions with a high prevalence of malaria and severe bacterial infections. The Chinese guideline of management of HIV/AIDS also recommends cotrimoxazole prophylaxis of CD4 < 200 cells/mm^3^ until CD4 > 200 cells/mm^3^ is achieved for longer than 3 months. Pre-emptive TB treatments are advised for any CD4 cell count. Additionally, fluconazole pre-emptive treatment in CrAg-positive adolescents and adults is recommended; however, screening is not advised for children [57].

## 6. Considerations for the Use of ART for Late Presenters

Asian guidelines generally do not differentiate between late and early presenters for ART [55, 56]. ART is recommended for all HIV-infected individuals irrespective of their CD4 cell count [55, 57], as studies have shown that ART initiation within one month significantly slows disease progression and improves immune recovery [64–67]. This is because the magnitude of CD4 recovery is directly correlated with starting CD4 cell count and ART initiation [68, 69]. Individuals have a better recovery rate and lower mortality if they initiate ART at higher CD4 threshold counts [68–70]. As such, adults with HIV, irrespective of their CD4 cell count, are recommended to initiate ART as soon as possible [52]. Same-day or rapid ART initiation (within seven days of diagnosis) is recommended for advanced HIV disease [57]; however, several individual patient considerations, such as coinfections (discussed above), should be taken into account prior to initiating ART. Bone mineral density testing is recommended for at-risk populations such as postmenopausal women, older men, and individuals with a history of fractures or hypogonadism [55]. Dolutegravir- (DTG-) based regimens were initially not advised for pregnant women or those looking to conceive [55, 71] because preliminary data indicated an increased risk of neural tube defects in infants born to those receiving DTG at the time of conception [72, 73]. However, updated results from the same study [74] showed lower prevalence of neural tube defects following DTG exposure than initial estimates; neural tube defect prevalence in infants born to women on DTG at conception was nonsignificantly higher than any other non-DGT antiretrovirals (0.06% difference; 95% CI 0.03–0.20) [74]. As such, the latest guidelines now consider DTG a recommended option for individuals of childbearing potential [75]. Pregnancy testing should be performed if there is a possibility of pregnancy; although ART is recommended for pregnant women, individuals should be counselled on the risk of the ART drugs during pregnancy. Genotypic resistance testing is also recommended for treatment-naive individuals or before a change of treatment due to treatment failure [55]. An HLA-B*∗*5701 test is recommended before prescribing abacavir as there is an increased risk of hypersensitivity reactions [56, 76].

Challenges to treatment adherence are thought to be one of the main reasons for poor HIV treatment outcomes [77]. Ongoing drug use presents a particular challenge in the treatment of HIV as it can impact treatment adherence and viral suppression [78, 79]. In addition to ART, late presenters, particularly those with a CD4 count below 200 cells/mm^3^, are often prescribed additional medicines to treat or prevent opportunistic infections. This presents a significant pill burden that can compromise treatment adherence [77, 80]. As such, the WHO recommends an intensified adherence support program which includes tailored counselling to support treatment literacy and home visits (where feasible) to boost treatment adherence [57]. Emerging ART therapeutics are being formulated as combination tablets in order to reduce individuals' pill burden [56].

Regimens containing protease inhibitors are commonly used in individuals with advanced HIV; however, integrase inhibitor (INI)-based regimens are being increasingly utilised for this population. INIs can rapidly reduce the viral load and appear to confer fewer side effects compared to other regimens [81]. INI-based regimens have been shown to be equally effective as PI-based regimens in patients with low CD4 cell counts and/or an AIDS-defining disease; there were no significant differences in discontinuation rates or virological response following 48 weeks of treatment [82]. Chinese specialists are increasingly using INIs for late presenters.

PLWH often suffer from premature age-related noninfectious comorbidities than the general population [83]. People with HIV are at a disproportionate risk of developing comorbidities such as chronic kidney disease and heard disease, commonly caused by chronic immune activation [84]. Comorbidities in PLWH must be addressed and managed simultaneously with the virus, which still represents a significant challenge [84].

## 7. Specific Responses to the Problem of Late Diagnosis

Future research efforts are needed to ascertain in-depth information on HIV late presentation across Asia since evidence from the international literature may not be fully relevant due to sociocultural and healthcare system differences. More data on sites of testing, sites of exposure, and the effectiveness of testing programs targeting late presenters (e.g., self-testing programs) will aid in the development of policies and programs aimed at reducing late presentation. Furthermore, there is a need for modelling studies that evaluate whether increases in the proportion of late presenters in annual incident HIV diagnoses is due to an increase in true late diagnosis or due to improved testing campaigns which increase detection in people who were infected long ago but only recently diagnosed.

Factors that would likely significantly reduce the problem of late diagnosis include increasing community education and awareness of HIV testing, reducing the stigma around HIV, and normalising HIV testing as an aspect of general health screening. Reducing structural stigma and discrimination would reduce barriers for individuals to knowing their own HIV status. As for normalising HIV testing, risk-based testing needs to move to universal or opt-out testing in areas with high prevalence to increase the early diagnosis proportion of HIV infection among female, heterosexual, or old patients [85]. HIV indicator conditions will also be helpful in decreasing late presentation [86, 87].

Publication of HIV testing guidelines that are specific to local settings is critical as differences in sociocultural and healthcare systems significantly impact the uptake of early testing. Anonymous testing contributes to early HIV testing and medical care [88], therefore increasing access to anonymous HIV test sites is likely to play a significant role in reducing late presentation, particularly in regions where routine testing is stigmatised and individuals have privacy and confidentiality concerns.

## 8. Conclusion

Early HIV diagnosis and treatment is the key to maximising the individual's benefit from ART and improving public health. Despite efforts to increase testing and care across Asia, an estimated 34–72% of people do not present for care until their CD4 cell count has decreased below 350 cells/mm^3^ or present with an AIDS-defining event [7, 8, 10]. Late presenters generally have a higher disease burden and higher morbidity and mortality. Late presentation also increases the public health burden, with evidence that increases the risk of community transmission and increases healthcare costs. Effective strategies are needed to reduce the incidence of late presentation across Asia. Initiatives focusing on community and healthcare provider HIV education, as well as the destigmatisation of HIV, will likely reduce the incidence of late presentation.

## Data Availability

No original data were presented in this review.
